# Embryonic ethanol exposure disrupts craniofacial neuromuscular integration in zebrafish larvae

**DOI:** 10.3389/fphys.2023.1131075

**Published:** 2023-02-07

**Authors:** Ritika Ghosal, Gissela Borrego-Soto, Johann K. Eberhart

**Affiliations:** Department of Molecular Biosciences, College of Natural Sciences and Waggoner Center for Alcohol and Addiction Research, University of Texas at Austin, Austin, TX, United States

**Keywords:** ethanol, cranial nerves, neuromuscular junction, motor neuron, teratogen, craniofacial

## Abstract

Forming a vertebrate head involves the meticulous integration of multiple tissue types during development. Prenatal alcohol exposure is known to cause a variety of birth defects, especially to tissues in the vertebrate head. However, a systematic analysis of coordinated defects across tissues in the head is lacking. Here, we delineate the effects of ethanol on individual tissue types and their integration during craniofacial development. We found that exposure to 1% ethanol induced ectopic cranial muscle and nerve defects with only slight effects on skeletal pattern. Ectopic muscles were, however, unaccompanied by ectopic tendons and could be partially rescued by anesthetizing the larvae before muscle fibers appeared. This finding suggests that the ectopic muscles result from fiber detachment and are not due to an underlying muscle patterning defect. Interestingly, immobilization did not rescue the nerve defects, thus ethanol has an independent effect on each tissue even though they are linked in developmental time and space. Time-course experiments demonstrated an increase in nerve defects with ethanol exposure between 48hpf-4dpf. Time-lapse imaging confirmed the absence of nerve pathfinding or misrouting defects until 48hpf. These results indicate that ethanol-induced nerve defects occur at the time of muscle innervation and after musculoskeletal patterning. Further, we investigated the effect of ethanol on the neuromuscular junctions of the craniofacial muscles and found a reduced number of postsynaptic receptors with no significant effect on the presynaptic terminals. Our study shows that craniofacial soft tissues are particularly susceptible to ethanol-induced damage and that these defects appear independent from one another. Thus, the effects of ethanol on the vertebrate head appear highly pleiotropic.

## 1 Introduction

Forming the vertebrate craniofacial complex is an intricate developmental process. Spatial patterning of numerous tissue components and their integration into one-another is critical to form a functional head. During the development of head, tissues of different origin are required to integrate with one another. For instance, mesoderm-derived muscle must attach to the craniofacial skeleton *via* tendon, both predominantly neural crest-derived (Olsson et al., 2005; Trainor, 2010). These muscles must, in turn, receive innervation from cranial motor neurons to coordinate movement of the facial structures (Chandrasekhar, 2004; Sudiwala and Knox, 2019). The presence of this wide range of tissue types and their complex anatomical organization makes the craniofacial region highly sensitive to genetic and environmental insult (Brent, 2004; Reynolds et al., 2011; Nishimura and Kurosawa, 2022).

Ethanol is a known teratogen that can cause numerous congenital malformations (Muralidharan et al., 2013). Reflecting craniofacial complexity, ethanol-induced developmental defects are particularly concentrated in the head (Petrelli et al., 2018). Affected individuals may display a range of concomitant developmental defects including craniofacial dysmorphology, sensory and motor debilities, as well as cognitive and behavioral impairments. These disorders are collectively termed ‘Fetal alcohol spectrum disorder’ or FASD. It is estimated that 1.1%–5% of children born in the US are affected by FASD (May et al., 2014; May et al., 2018). The most serious manifestation of this condition is Fetal Alcohol Syndrome (FAS) which results in a combination of neurological and structural defects (Muggli et al., 2017; Brown et al., 2019).

The brain is the most commonly and the most severely affected organ in FASD (Reynolds et al., 2011). The consequence of alcohol-induced damage in the brain can manifest in a range of cognitive and neurobehavioral disorders affecting general intelligence, speech, swallowing, learning and memory among others (Denny et al., 2017; Wilhoit et al., 2017; Blanck-Lubarsch et al., 2019; Mattson et al., 2019). While many of the behavioral disorders are linked to cognition due to brain damage. They can also be caused due to structural and integration defects in the muscles, their attachments, or their innervation that can cause disruption in neuromuscular transmission (Lee et al., 2011; Lutz et al., 2020). However, the effect of ethanol on muscle integration and innervation in the face is not clearly understood.

Here, we have carried out a systematic study to examine the effect of embryonic ethanol exposure on the anatomical organization and integration of the craniofacial complex. We first studied the effects of ethanol exposure on each individual tissue component, the craniofacial skeleton, musculature, connective tissues and motor nerves. This enabled us to compare the dose-specific effects on severity and frequency of structural defects induced on each individual tissue type. We then determined if there were associations between ethanol-induced defects in different tissue components. We found that ethanol adversely effects soft tissue integration and disrupts the neuromuscular junction which might be linked to the motor skill deficits induced in FASD.

## 2 Results

### 2.1 Ethanol can cause mild defects to the branchial skeleton

The vertebrate craniofacial complex houses the brain, head sensory organs, the feeding, and the respiratory apparatus, among other organs. Assembling the craniofacial complex is therefore an arduous developmental event. We sought to understand if and how ethanol disrupts the integration of the vertebrate craniofacial complex. We used zebrafish due to the abundance of transgenic lines allowing for visualization of different tissue components in the developing head.

**FIGURE 1:**
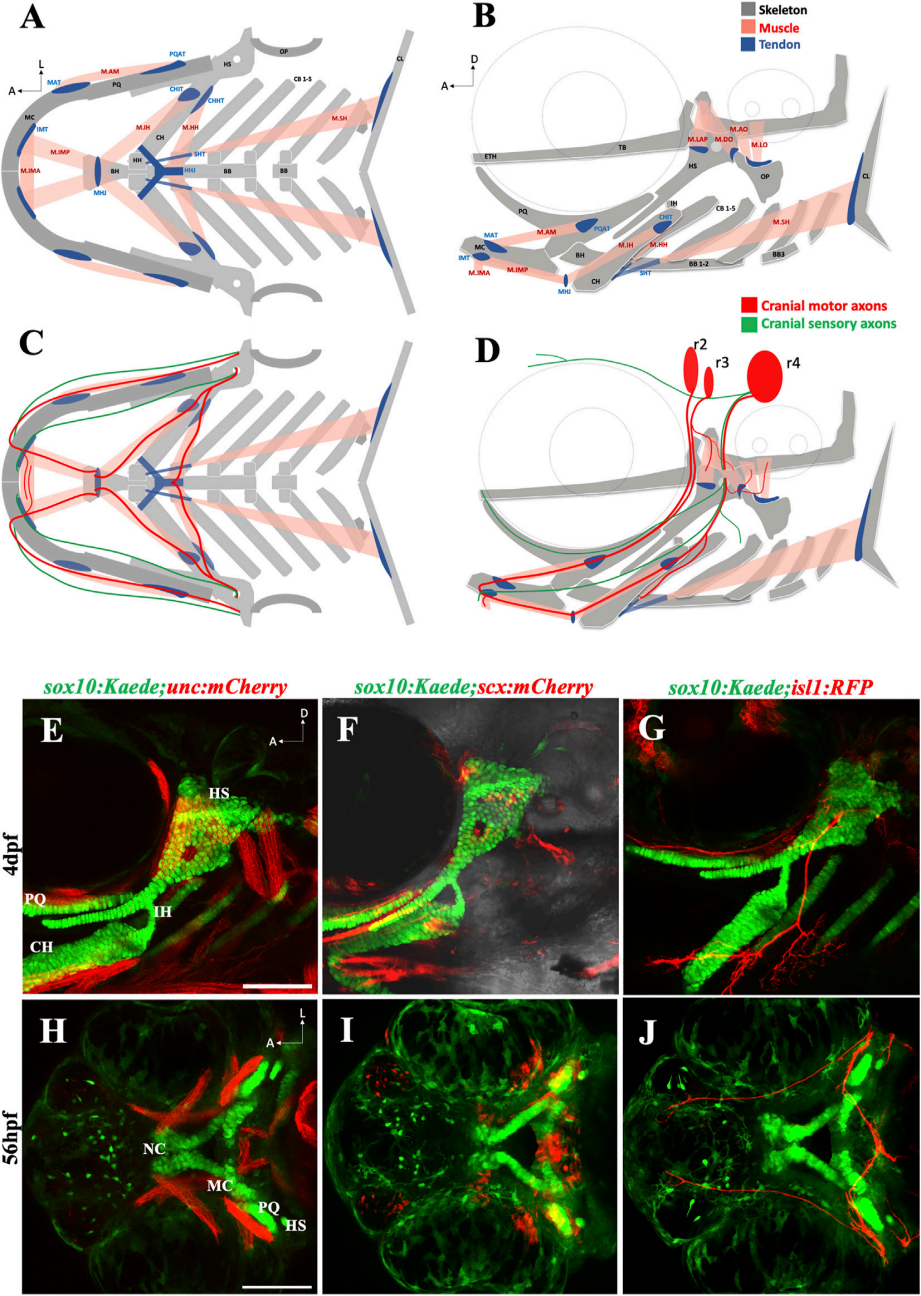
The zebrafish head has a complex anatomical organization **(A, B)** Schematics showing arrangement of craniofacial skeleton, muscle, and tendon in ventral and lateral planes (adapted from Schilling and Kimmel, 1997). **(C, D)** Schematics showing cranial nerves originating from rhombomere (r) 2, 3 & 4 innervating the branchial muscles in ventral and lateral planes. **(E)** 4dpf *sox10:Kaede;unc:mCherry* double transgenic fish showing musculoskeletal arrangement laterally. **(F)** 4dpf *sox10:Kaede;scx:mCherry* double transgenic fish showing tendons and cartilage laterally. **(G)** 4dpf *sox10:Kaede;isl1:RFP* double transgenic fish showing branchiomotor neurons and cartilage laterally. **(H–J)** Ventral view of developing zebrafish head at 56hpf showing craniofacial cartilage, muscle, tendons, and motor neurons emerge and integrate around the same developmental window. A (anterior), D (dorsal) L (lateral). r (rhombomere). Skeleton (grey)—BB (basibranchial), BH (basihyal), CB (ceratobranchial), CH (ceratohyal), CL (cleithrum), ETH (ethmoid), TB (trabecula), HH (hypohyal), HS (hyosymplectic), MC (Meckel’s cartilage), OP (opercle), PQ (palatoquadrate). Tendon (blue)—CHHT (ceratohyal hyohyoideus tendon), CHIT (ceratohyal interhyoideus tendon), HHJ (hyohyal junction), IMT (intermandibular tendon), MAT (Meckel’s adductor tendon), MHJ (mylohyoid junction), PQAT (palatoquadrate adductor tendon), SHT (sternohyoideus tendon). Muscle (pink)—M.AM (adductor mandibularis muscle), M. AO (adductor operculi muscle), M. DO (dilator operculi muscle), M. HH (hyohyoideus muscle), M. IH (interhyoideus muscle), M. IMA (Intermandibularis anterior muscle), M. IMP (Intermandibularis posterior muscle), M. LAP (levator arcus palatini muscle), M. LO (levator operculi muscle), M. SH (sternohyoideus muscle). Scale bar = 100 um.

Like other vertebrates, the zebrafish craniofacial complex has an intricate anatomical organization (Figures 1A–D). We focused on the oropharyngeal apparatus to investigate the effect of ethanol on craniofacial integration. We used a set of transgenic zebrafish lines to visualize individual tissue components: skeleton, muscle, connective tissue, and the motor neurons innervating the oropharyngeal (branchial) muscles (Figures 1E–G). These tissue types emerge and get assembled around the same developmental time window and are in close proximity to one another (Figures 1H–J). Thus, ethanol-exposure even for a short duration can potentially damage one or more of these concurrently developing tissues contributing to the heterogeneity of FASD craniofacial phenotype.

**FIGURE 2:**
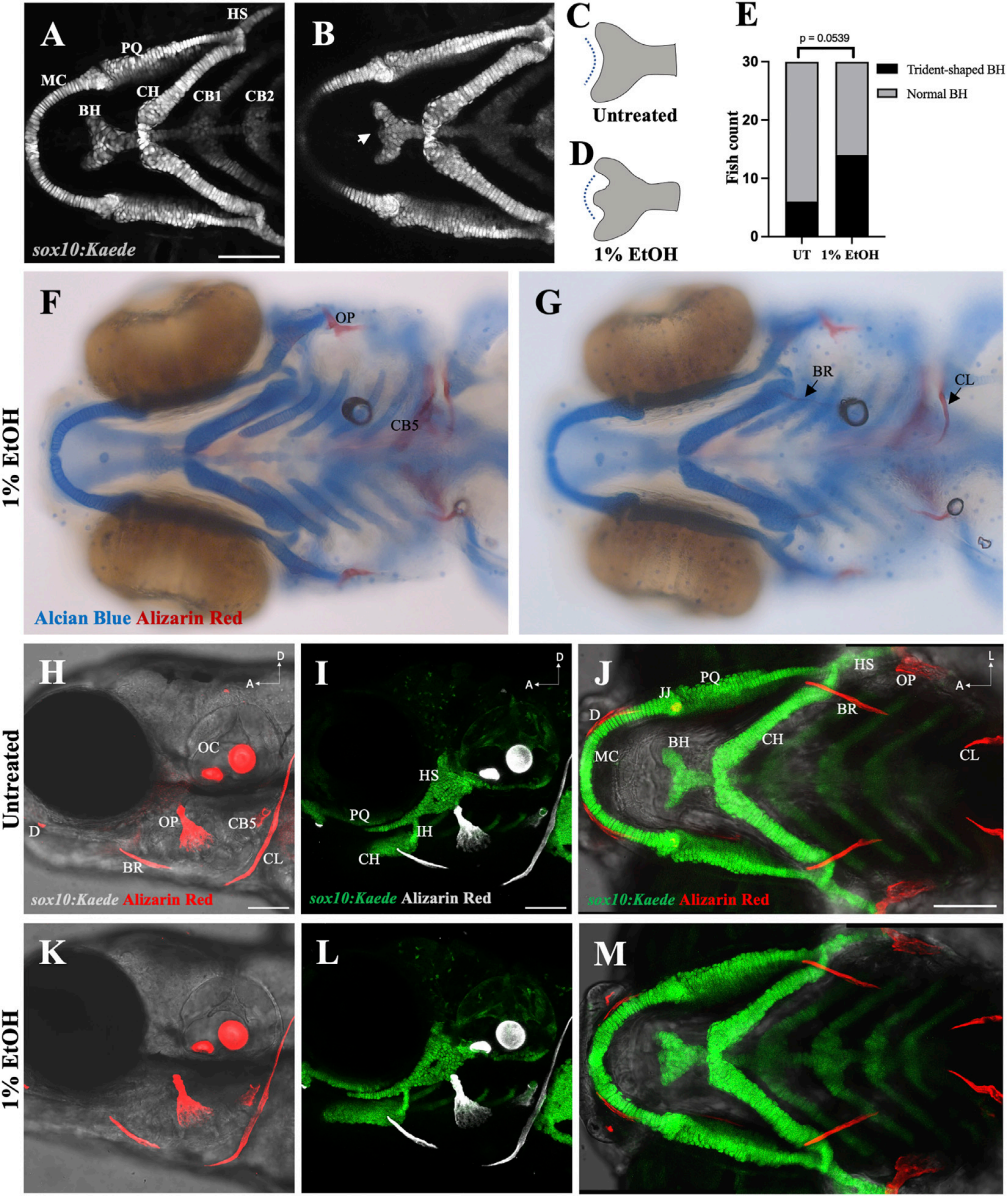
Ethanol can have mild effects on the shape of the basihyal cartilage **(A)** Ventral view of branchial cartilages in untreated (UT) *sox10:Kaede* fish at 4dpf. **(B)** Ventral view of branchial cartilages in ethanol (EtOH)-exposed *sox10:Kaede* fish at 4dpf showing trident-shaped basihyal (indicated by an arrow). **(C, D)** Schematics showing basihyal shape in untreated **(C)** and ethanol-exposed fish **(D)**. **(E)** Number of fish with trident-shaped basihyal increase in the ethanol-exposed group. **(F, G)** Alcian and alizarin-stained, ethanol-exposed viscerocranium showing normal ossification at 4dpf in AB fish. **(H–J)** Live alizarin-stained untreated *sox10:Kaede* fish at 4dpf. **(K–M)** Live alizarin-stained *sox10:Kaede*, ethanol-exposed fish at 4dpf showing no ossification defects. BR (branchiostegal ray), CB5 (ceratobranchial 5), CL (cleithrum), D (dentary), JJ (jaw joint), OC (otic capsule), OP (opercle). Scale bar = 100 um.

Previous study from our laboratory using cartilage and bone double staining had indicated that exposure to 1% ethanol does not affect skeletogenesis. We reinvestigated this using the *sox10:Kaede* and *sox10:mRFP* transgenic lines to visualize the branchial cartilages. Consistent with previous findings, cartilage development was largely normal in zebrafish exposed to 1% ethanol from 6hpf (hours post fertilization) to 4dpf (days post fertilization). However, ethanol did alter the shape of the basihyal cartilage, from a normally concave shape to a trident-shape (Figure 2B). We scored the number of *sox10:Kaede* fish with trident-shaped basihyal in ethanol-exposed and untreated fish at 4dpf, with *n* = 30 for each group. We found that 46.67% (14/30) ethanol-exposed fish displayed a trident-shaped basihyal as opposed to 20.0% (6/30) in the untreated group. This difference fell just outside statistical significance based on Fisher’s exact test of independence (p = 0.0539). However, this odds ratio of 2.335 suggests that ethanol elevates the risk of potentially biologically significant changes in cartilage morphology.

We also investigated the effect of 1% ethanol exposure on branchial ossification using alizarin-red staining. Our investigation did not reveal any observable structural defect in the dermal bones of the branchial skeleton at 4dpf (Figures 2F–M). These results demonstrate that exposure to 1% ethanol at gastrulation causes few, if any, cranial skeletal defects on 4dpf zebrafish.

### 2.2 Ethanol induces ectopic cranial muscle

**FIGURE 3:**
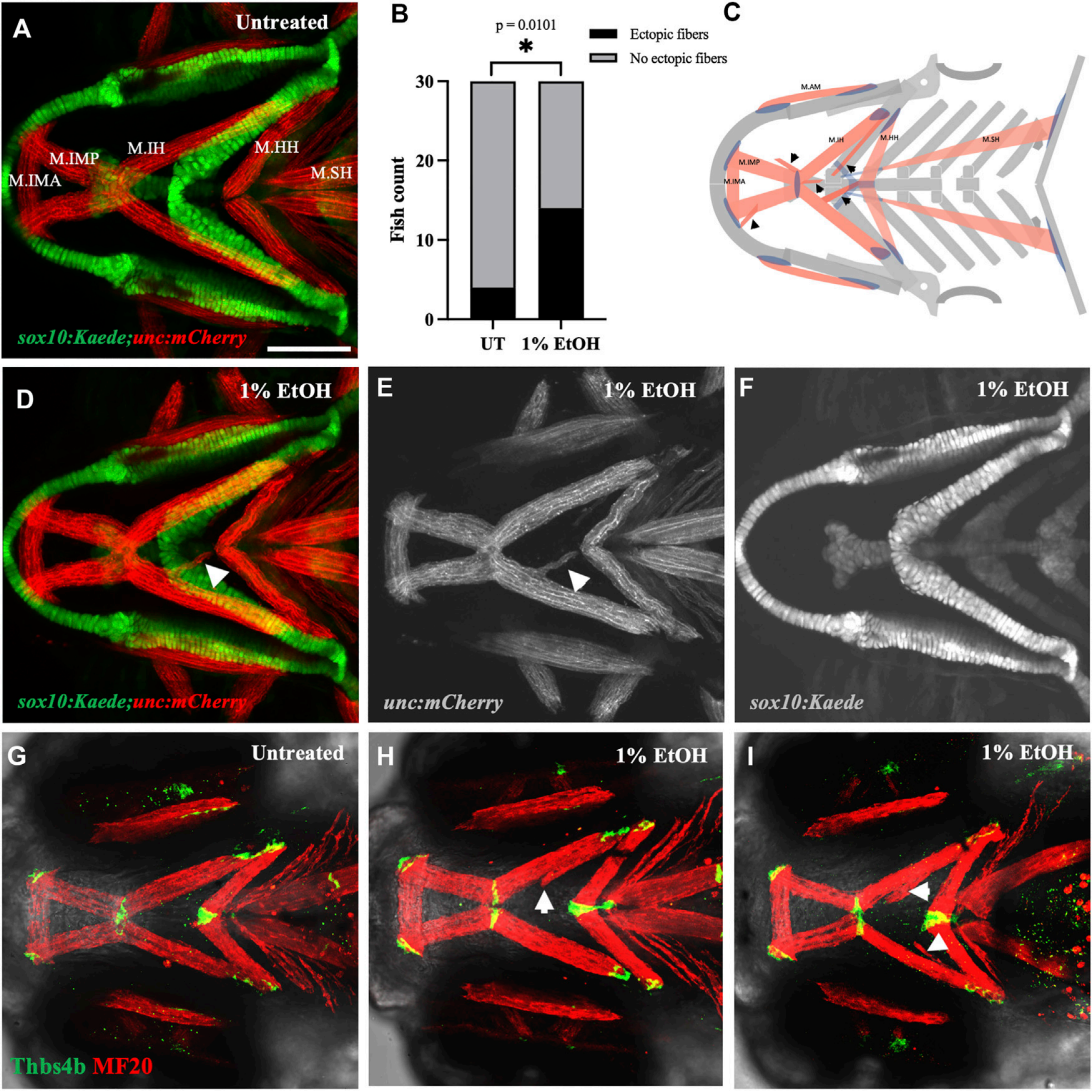
Ethanol induces ectopic muscle fibers **(A)** Ventral view of branchial skeleton and muscle in untreated *sox10:Kaede;unc:mCherry* fish at 4dpf. **(D–F)** Ethanol-exposed *sox10:Kaede;unc:mCherry* fish at 4dpf. Arrows show ectopic muscle fiber in D & E. **(B)** Ectopic muscle fibers significantly increase in the ethanol-exposed fish. **(C)** Schematic showing locations of ectopic muscle fiber defects observed in the ethanol-exposed fish. **(G)** Untreated *isl1:RFP* fish immunolabeled with MF20 (heavy chain of myosin II) in red and Thbs4b (Thrombospondin 4 b) in green. **(H, I)** MF20 & Thbs4b immunolabeled, ethanol-exposed *isl1:RFP* fish. Arrows show ectopic muscle fibers lack ectopic tendons. * = *p* < 0.05. Scale bar = 100 um.

We used the *sox10:Kaede;unc:mCherry* double transgenic line to label the branchial skeleton and muscles. Embryos exposed to 1% ethanol from 6hpf to 4dpf had ectopic muscles at 4dpf despite an intact skeletal pattern (Figures 3D–F). Few ectopic muscle fibers were present in unexposed zebrafish (4/30, 13.33%), this proportion was greatly elevated in fish exposed to ethanol (14/30, 46.67%). A Fisher’s exact test indicated there was a significant increase in the number of fish with ectopic muscles (Figure 3B, p = 0.0101). The ectopic cranial muscle fibers were observed predominantly around the Intermandibularis posterior and Interhyal muscles (Figure 3C). We did not find any significant increase in the number of *unc:mCherry* fish with ectopic muscles at ethanol doses below 1% (data not shown).

The musculoskeletal patterning in the pharyngeal arches is primarily regulated by the cranial neural crest cells (CNCs). The organization of the branchial muscles is driven by the CNC-derived cranial connective tissues (Rinon et al., 2007; Trainor, 2010; McGurk et al., 2017; Schneider, 2018; Ziermann et al., 2018). We, therefore, examined if the ectopic muscle fibers were associated with ectopic tendons. Using whole mount immunohistochemistry, we colabeled the ethanol-exposed 4dpf zebrafish with muscle and tendon markers. Interestingly, we found that the ectopic muscle fibers in the ethanol-exposed fish were not associated with ectopic tendons (Figures 3H, I). The lack of tenocytes associating with the ectopic muscle fibers suggests the defect is not in the genesis of musculoskeletal patterning. Instead, these ectopic muscles may have detached from their original attachment site.

A detachment would require force. Therefore, we tested this possibility by anaesthetizing the ethanol-exposed fish in MESAB (MS 2222/Tricaine) to restrict muscle movement. The branchial muscles in zebrafish emerge after 52hpf (Schilling and Kimmel, 1997). Ethanol-exposed embryos were treated with 0.075 mg/mL MESAB at 52hpf to reduce movement during the extension and attachment of branchial muscles. Again, we found that ethanol caused a significant increase in ectopic muscle fibers, with 60% (9/15) of ethanol-exposed non-anaesthetized fish and 13.33% (2/15) of unexposed fish having ectopic fibers (*p* = 0.0209). Anesthetic exposure reduced the number of fish with ectopic fibers to 26.67% (4/15) (Supplementary Figure S1), intermediate to, and not significantly different from, either exposed or unexposed fish. Restricting muscle movement, thus, partially rescued the ectopic muscle phenotype in the ethanol-exposed fish. This confirms that the ethanol-induced ectopic muscle fibers resulted, at least in part, from detachment.

### 2.3 Ethanol can cause mis-location of the mylohyoid junction

**FIGURE 4:**
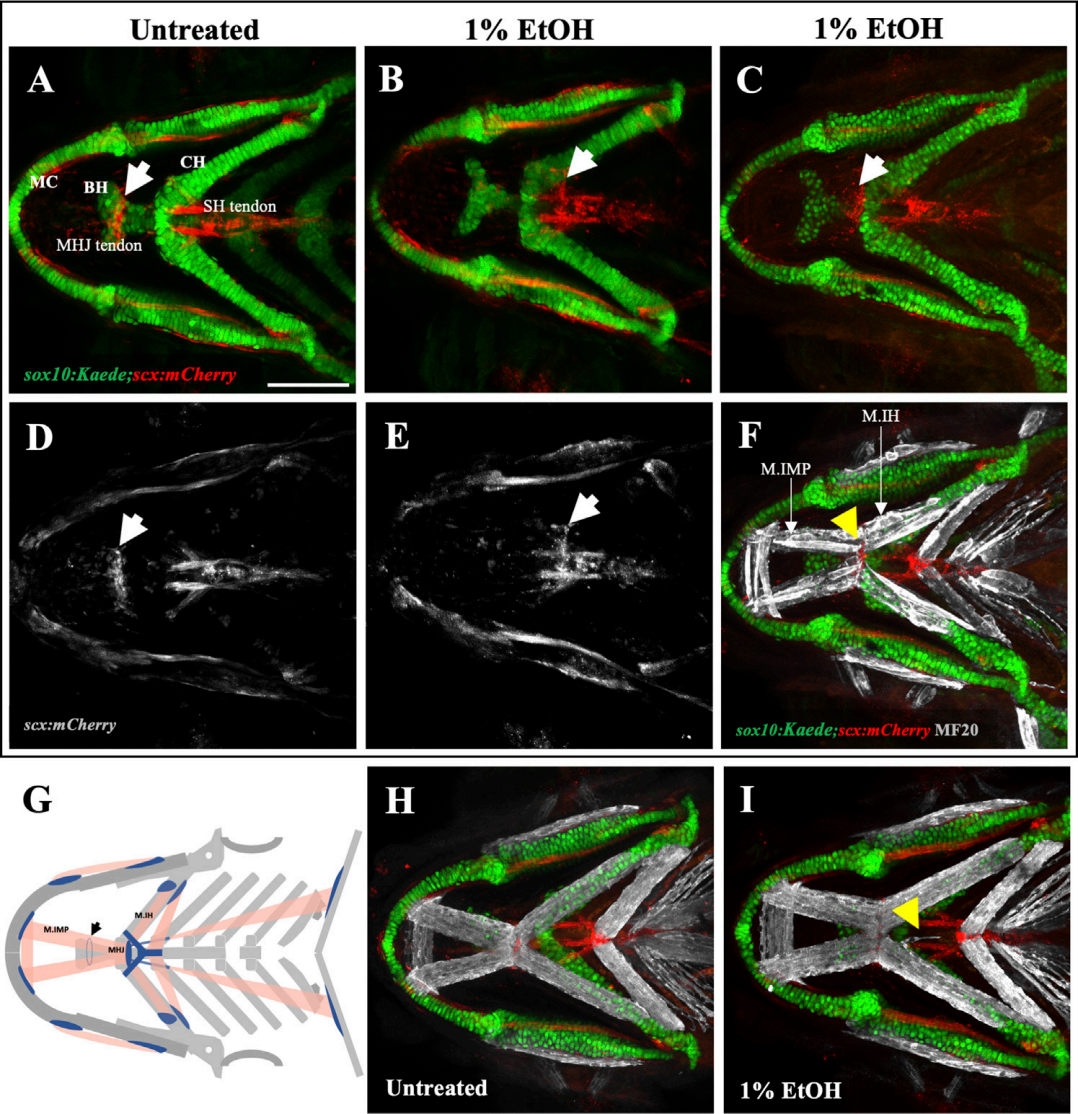
Ethanol can mislocate myotendinous attachment tissues **(A, D)** Ventral view of branchial cartilage and tendon in untreated *sox10:Kaede;scx:mCherry* fish at 4dpf. Arrows show the normal position of the MHJ (mylohyoid junction) around the anterior basihyal. **(B,C, E)** Posteriorized MHJ position in ethanol-exposed *sox10:Kaede;scx:mCherry* fish at the level of SHT (sternohyoideus tendon) in **(B, E)** and at the anterior end of ceratohyal in **(C) (F)** Ethanol-exposed *sox10:Kaede;scx:mCherry* fish immunostained with MF20 (gray), anti-Kaede (green) and anti-mCherry (red) antibodies. Arrow shows that the posterior end of the Intermandibularis posterior muscle (M.IMP) aligns with the MHJ tendon and is located at the level of ceratohyal. **(G)** Schematic showing posteriorized MHJ tendon and Intermandibularis posterior muscle (M.IMP). Arrow indicates the normal position of the MHJ tendon. **(H, I)** Untreated **(H)** and ethanol-exposed **(I)** *sox10:Kaede;scx:mCherry* fish immunolabeled with MF20 (gray), anti-Kaede (green) and anti-mCherry (red) antibodies. Arrow in I shows ectopic muscle fiber without any effect on tendon pattern. Scale bar = 100 um.

While ectopic muscle fibers were not accompanied by ectopic tendons, it was not clear whether ethanol has an overall effect on tendon morphology. We investigated this using *sox10:Kaede;scx:mCherry* double transgenics to visualize the branchial skeleton and tendons. We exposed the embryos to 1% ethanol from 6hpf to 4dpf and analyzed them for tendon defects at 4dpf. We found that the myotendinous attachment between the Intermandibularis posterior muscle and the Interhyal muscle at the mylohyoid junction was moved posterior in the ethanol-exposed fish (*n* = 2 out of the 30 fish analyzed). Normally, the mylohyoid junction (MHJ) tendon is located towards the anterior end of the basihyal cartilage (Figures 4A, D). In ethanol-exposed fish, it was found at more posterior locations near the sternohyoid tendon (Figures 4B, E) and at the anterior end of the ceratohyal cartilage (Figure 4C). We did not observe any alterations in the positions of other branchial tendons in the ethanol-exposed fish. The untreated fish (*n* = 30) invariably did not display any visible tendon defects (Figure 4D).

The mylohyoid junction (MHJ) tendon is thought to be the only myotendinous attachment in the branchial region of the fish which does not have a skeletal attachment. All other tendons of the ventral branchial muscles are associated with a skeletal element. An orthogonal view through the ventral branchial region demonstrated a physical separation between the MHJ tendon and the basihyal cartilage (Supplementary Figure S2A). Conversely, orthogonal views through other branchial tendons for instance, the sternohyoid (SH) tendon that attaches the sternohyoid muscle to hypohyals showed a clear physical association between tendon and cartilage (Supplementary Figure S2B). 3-D rendering of a confocal Z-stack through the mylohyoid junction also demonstrated that the MHJ tendon is not attached to the basihyal cartilage (Supplementary Figure S2D; Movie1). These results confirm that zebrafish MHJ tendon at 4dpf does not have a skeletal attachment. Interestingly, ethanol seems to have an effect only on the MHJ tendon which uniquely is devoid of any hard tissue association.

Since the CNC-derived cranial tendons are known to organize the cranial muscle pattern. A mislocated MHJ tendon is expected to mislocate the posterior end of the Intermandibularis posterior muscle. To test this, we immunolabeled the ethanol-exposed, *sox10:Kaede;scx:mCherry* fish with a muscle marker. Expectedly, we found, the posterior end of the Intermandibularis posterior muscle aligned with the MHJ tendon in all fish. In an untreated fish, the posterior end of Intermandibularis posterior muscle is located around the anterior basihyal. However, in ethanol-exposed fish (Figure 4F) the posterior end of Intermandibularis posterior muscle was at the anterior end of ceratohyal, associated with the posteriorly localized MHJ. Thus, ethanol exposure can posteriorize the whole MHJ assembly.

Taken together these results show that ethanol can induce ectopic muscle fibers without affecting the skeleton or associated connective tissues (Figure 4I). Ethanol can, however, mislocate tendons not associated with the skeleton. This suggests that hard tissues are more resilient to ethanol-induced damage.

### 2.4 Ethanol induces cranial nerve defects

**FIGURE 5:**
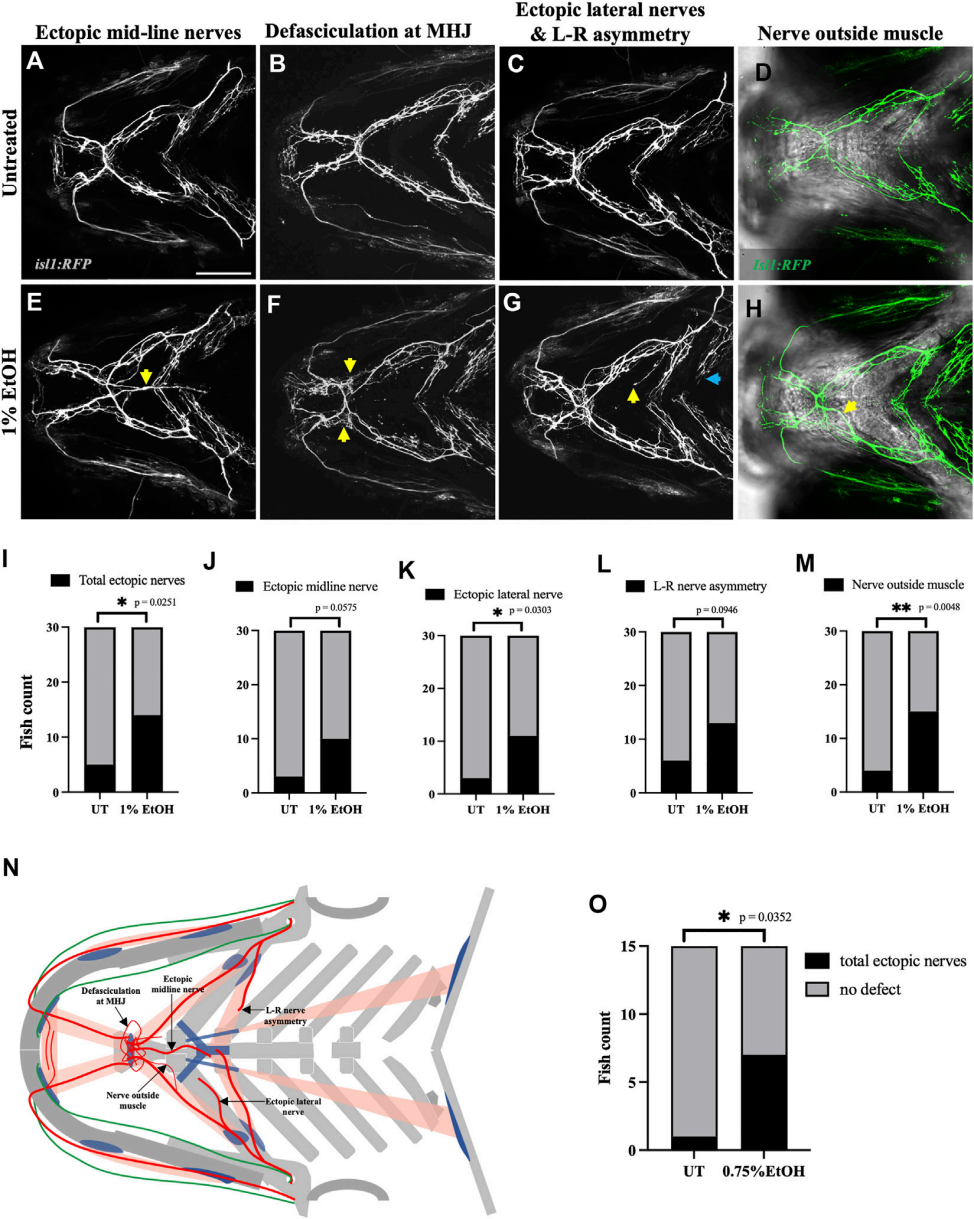
Ethanol induces cranial nerve defects **(A–D)** Untreated *isl1:RFP* fish showing motor innervation in the ventral branchial muscles at 4dpf. **(E–H)** Ethanol-exposed *isl1:RFP* fish showing various motor innervation defects. **(E)** Arrow shows ectopic nerves along the medial axis. **(F)** Arrows show defasciculation at mylohyoid junction (MHJ). **(G)** Yellow arrow shows ectopic nerve along the lateral axis. Blue arrow shows asymmetrical extension of nerves innervating the left and right side of the hyohyoideus muscle. **(H)** Maximum intensity Z-projected image with motor nerves (in green) merged with a single Z-plane of brightfield channel showing boundary of the interhyoideus muscle. Arrow indicates motor axons located outside the muscle boundary. **(I–M)** Number of fish with various cranial nerve defects in untreated and ethanol-exposed groups (*n* = 30 in each group). Number of fish with total ectopic nerves, ectopic lateral nerves and nerves outside the muscle boundary significantly increase in the ethanol-exposed group in I, K, M. **(N)** Schematic showing different cranial nerve defects found in the ethanol-exposed fish. **(O)** Number of fish with total ectopic cranial nerves significantly increase in 0.75% ethanol-exposed *isl1:RFP* fish (*n* = 15 in each group). * = *p* < 0.05; ** = *p* < 0.01. Scale bar = 100 um.

The vertebrate branchial muscles are innervated by the cranial motor neurons located in the hindbrain (Guthrie, 2007). To investigate the effect of ethanol on cranial nerves we used *isl1:GFP* and *isl1:RFP* transgenic lines which label the branchiomotor neurons. Exposing *isl1:RFP* embryos to 1% ethanol from 6hpf to 4dpf induced several different kinds of cranial nerve defects. We categorized these defects into five groups: 1) Ectopic midline nerves-ectopic axonal fibers along the medial axis (Figure 5E), 2) Defasciculation- Nerve bundling defects particularly at the mylohyoid junction (MHJ) (Figure 5F), 3) Lateral ectopic nerves-ectopic nerves along the lateral axis (yellow arrows in Figure 5G), 4) Left-Right asymmetry-disproportionate nerve elongation in the left and right hyohyoideus muscle (blue arrows in Figure 5G), and 5) nerve outside muscle-mislocated nerves situated outside the muscle boundary (Figure 5H). We found a significant increase in the number of fish with total ectopic nerves, ectopic lateral nerves and mislocated nerves outside the muscle boundary (Figures 5I, K, M) in the ethanol-exposed group (*n* = 30) relative to the untreated group (*n* = 30). Although, the ectopic midline nerve defects were not significant in this analysis, however, the data shows a strong trend (Figure 5J).

Next, we investigated if the nerve defects can be induced by ethanol concentration below 1%. We found that exposing *isl1:RFP* embryos to 0.75% ethanol from 6hpf to 4dpf significantly increased the total ectopic nerve defects observed at 4dpf (Figure 5O). Thus, suggesting that motor neurons are more ethanol-sensitive than muscles as the latter were unaffected at ethanol concentrations below 1%.

**FIGURE 6:**
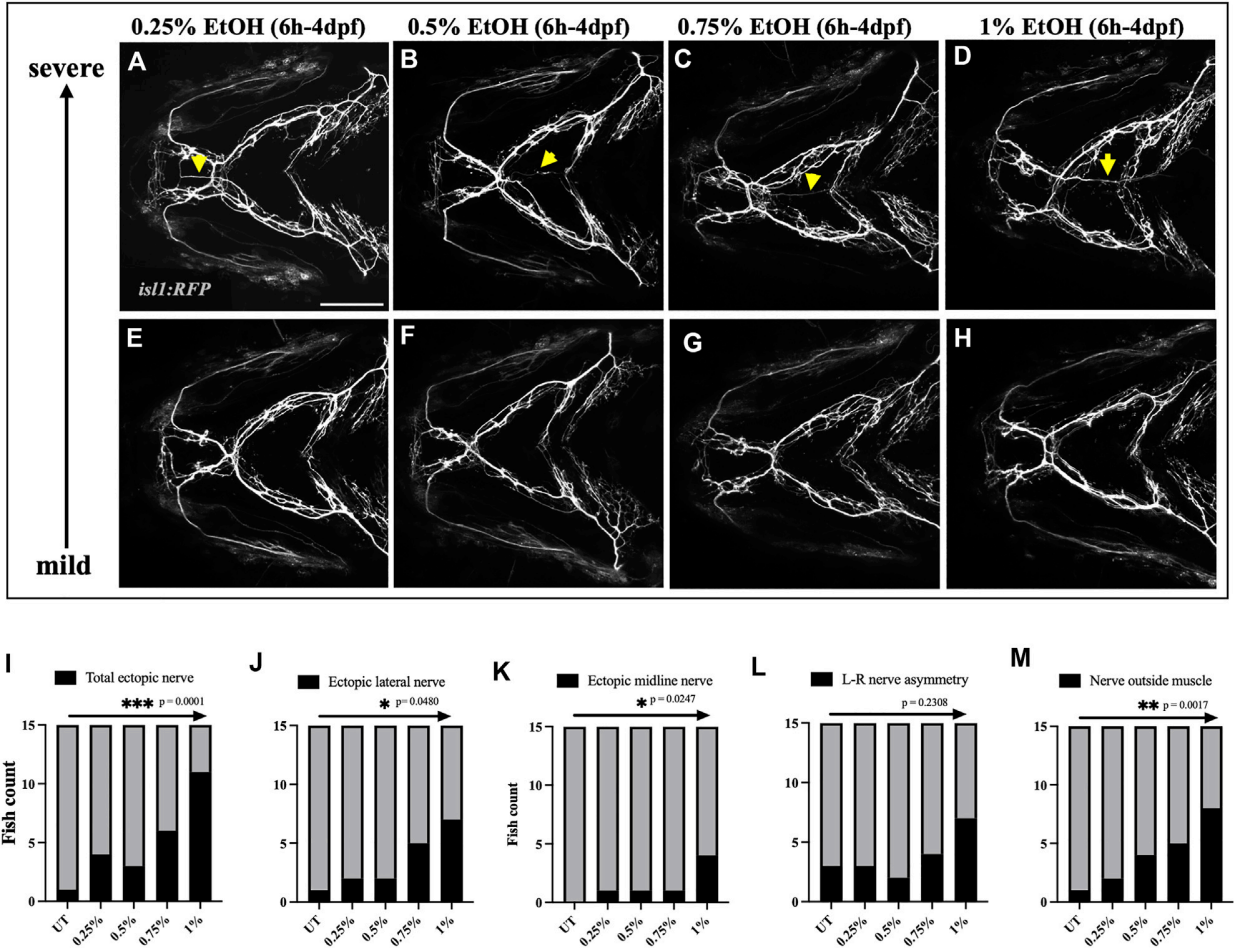
Prevalence of ectopic cranial nerve defects increases in a dose-dependent manner **(A–D)** Representative images of most severe cranial nerve defects in *isl1:RFP* fish exposed to 0.25%, 0.5%, 0.75% % 1% ethanol between 6hpf-4dpf (*n* = 15 in each group). Arrows indicate ectopic nerves. **(E–H)** Representative images of fish with mild cranial nerve defects in 0.25%, 0.5%, 0.75% % 1% ethanol-exposed groups. **(I–M)** Chi-square test for trend shows a linear trend between the number of fish with cranial nerve defects and the concentration of ethanol. * = *p* < 0.05; ** = *p* < 0.01; *** = *p* < 0.001. Scale bar = 100 um.

We then carried out a dose-response study to investigate if the frequency and severity of these nerve defects depend on the level of ethanol exposure. We exposed *isl1:RFP* embryos to 0.25%, 0.5%, 0.75% & 1% ethanol from 6hpf to 4dpf and characterized nerve defects at 4dpf (Figures 6A–H). Using the Chi-square test for trend, we found that the frequency of nerve defects increased in a concentration-dependent manner (Figures 6I–M). These results indicate that higher ethanol concentrations increase the prevalence of cranial nerve defects.

**FIGURE 7:**
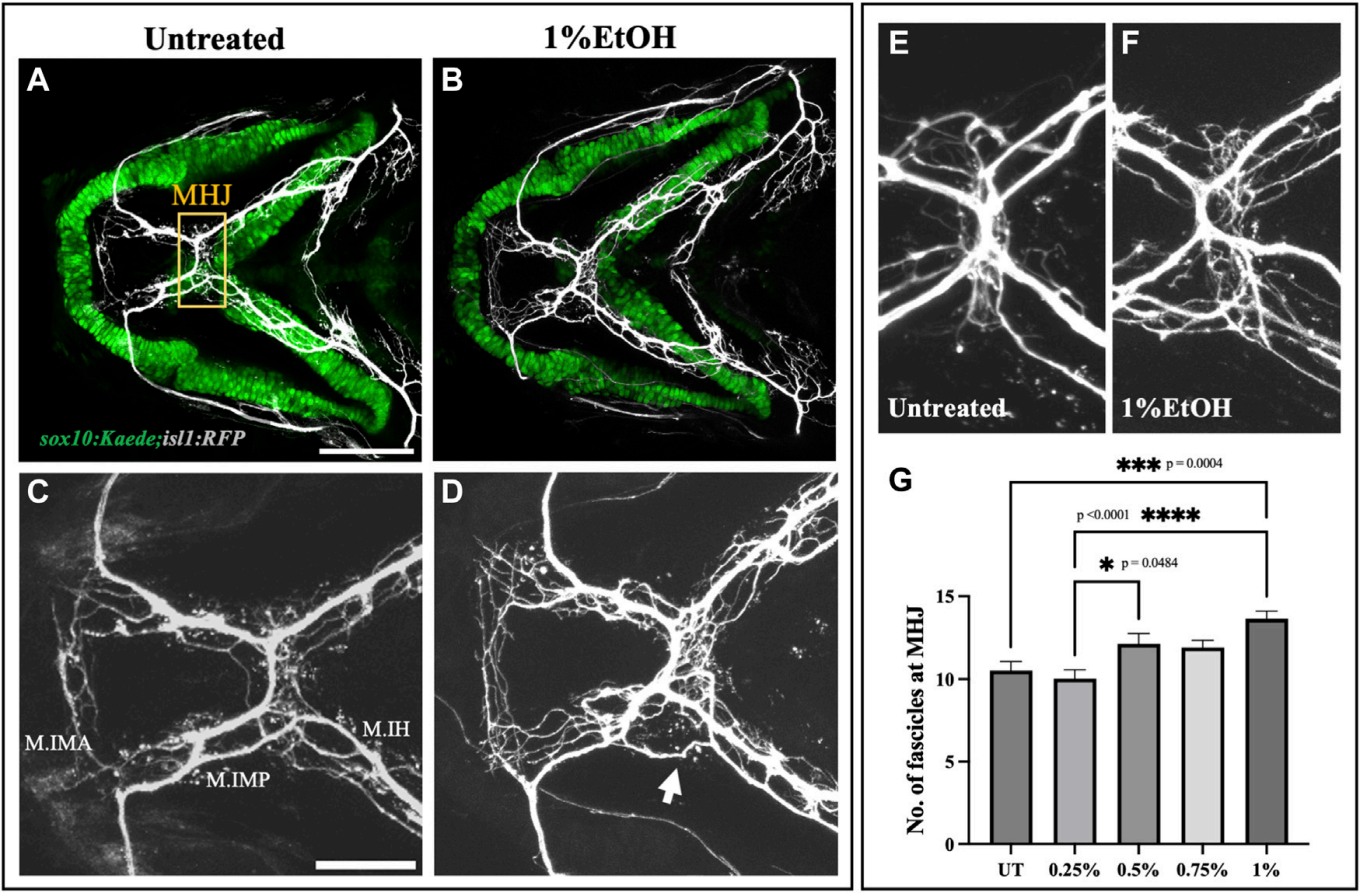
Ethanol induces cranial nerve defasciculation **(A, B)** Untreated and ethanol-exposed *sox10:Kaede;isl1:RFP* fish. Scale bar = 100 um. **(C, D)** Magnified view of motor innervations in Intermandibularis anterior (M.IMA), Intermandibularis posterior (M.IMP) and Interhyoideus muscles (M.IH) in untreated and ethanol-exposed fish. Scale bar = 50 um. Arrow in D indicates defasciculated exons around MHJ (mylohyoid junction). **(E, F)** High-resolution images of MHJ region in untreated and ethanol-exposed fish. **(G)** Number of fascicles at MHJ significantly increase in fish exposed to 1% ethanol.* = *p* < 0.05; *** = *p* < 0.001; **** = *p* < 0.0001.

We also examined the fasciculation at the mylohyoid junction in the ethanol-exposed fish (Figures 7B, D). We manually counted the number of fascicles at the MHJ to quantify defasciculation. We found a significant increase in the number of fascicles in fish exposed to 1% ethanol (Figure 7G, Supplementary Table S1). Suggesting, defasciculation in the cranial nerves can be induced by ethanol exposure. Together, these results show that the cranial nerves are highly susceptible to ethanol-induced defects and the frequency of nerve defects increases at higher ethanol concentrations.

### 2.5 Ethanol induces ectopic cranial nerves at the time of muscle innervation

**FIGURE 8:**
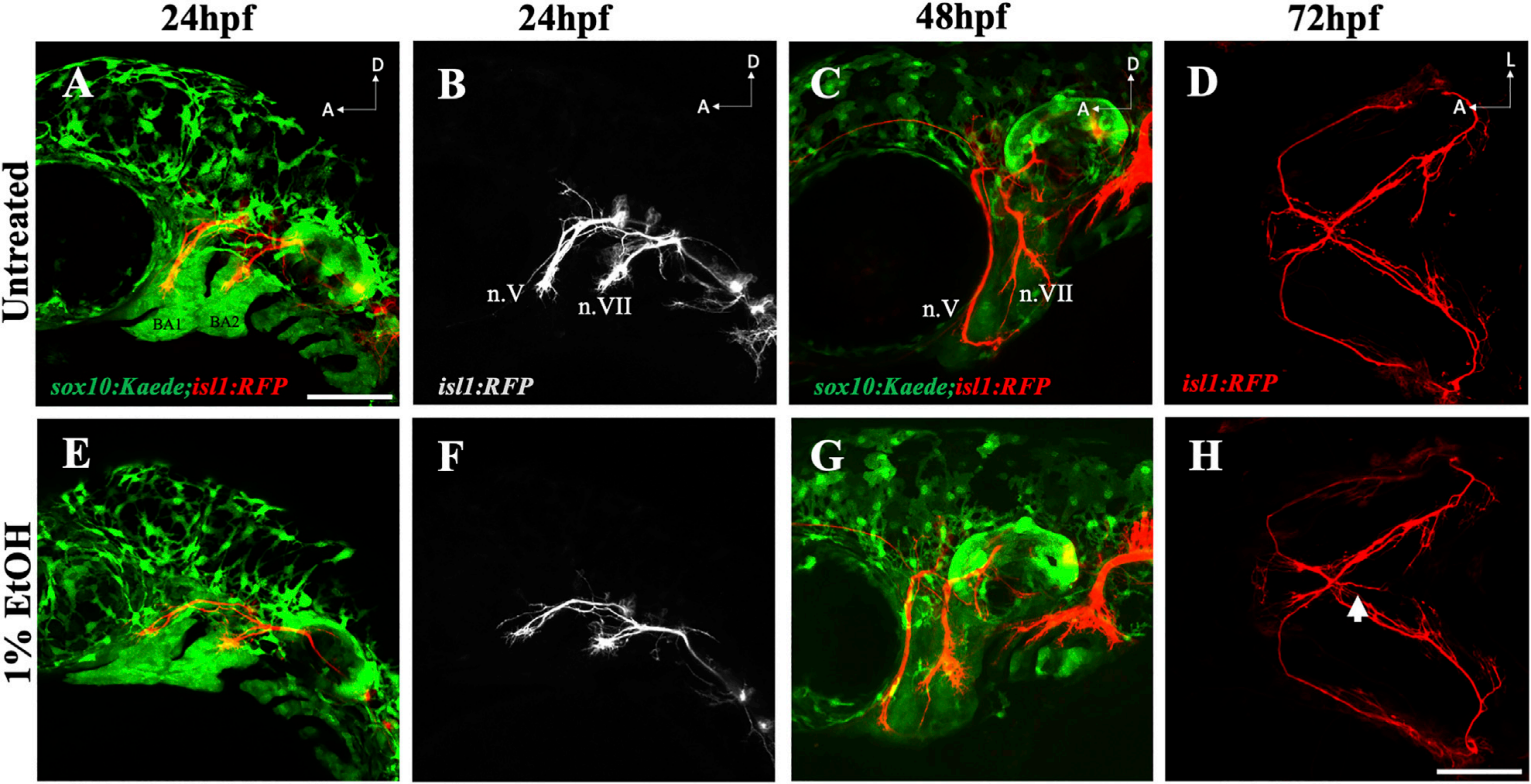
Ethanol does not affect early cranial nerve routing and pathfinding **(A, B)** Trigeminal nerve (n.V) and facial nerve (n.VII) extending towards BA (branchial arch)1 and BA2 in untreated *sox10:Kaede;isl1:RFP* embryo at 24hpf. **(E, F)** Normal extension of n. V and n. VII in ethanol-exposed *sox10:Kaede;isl1:RFP* embryos at 24hpf. **(C, G)** Untreated and ethanol-exposed *sox10:Kaede;isl1:RFP* fish at 48hpf. **(D, H)** Untreated and ethanol-exposed *isl1:RFP* fish at 72hpf. Arrow in H shows an extending ectopic nerve along the midline in ethanol-exposed fish. Scale bar = 100 um.

The mislocated ectopic nerves prompted us to examine if ethanol can induce misrouting or pathfinding defects in cranial nerves. Using the *sox10:Kaede;isl1:RFP* double transgenic line we investigated the early axonal projections of the trigeminal nerve (n.V) and the facial nerve (n.VII) extending towards the mandibular and the hyoid arch respectively. Time-lapse imaging from 24hpf-48hpf did not reveal any noticeable difference in the cranial nerve routing between ethanol-exposed and untreated embryos (Movie 2, 3). We did not find any observable mispositioning of nerve fibers in the ethanol-exposed embryos examined at 24hpf (*n* = 25) & 48hpf (*n* = 15) (Figures 8A–C, E–G). However, at 72hpf, we found the first appearance of the ectopic nerves along the midline (Figure 8H). These results suggest that ethanol-induced ectopic nerve defects are induced after 48hpf.

**FIGURE 9:**
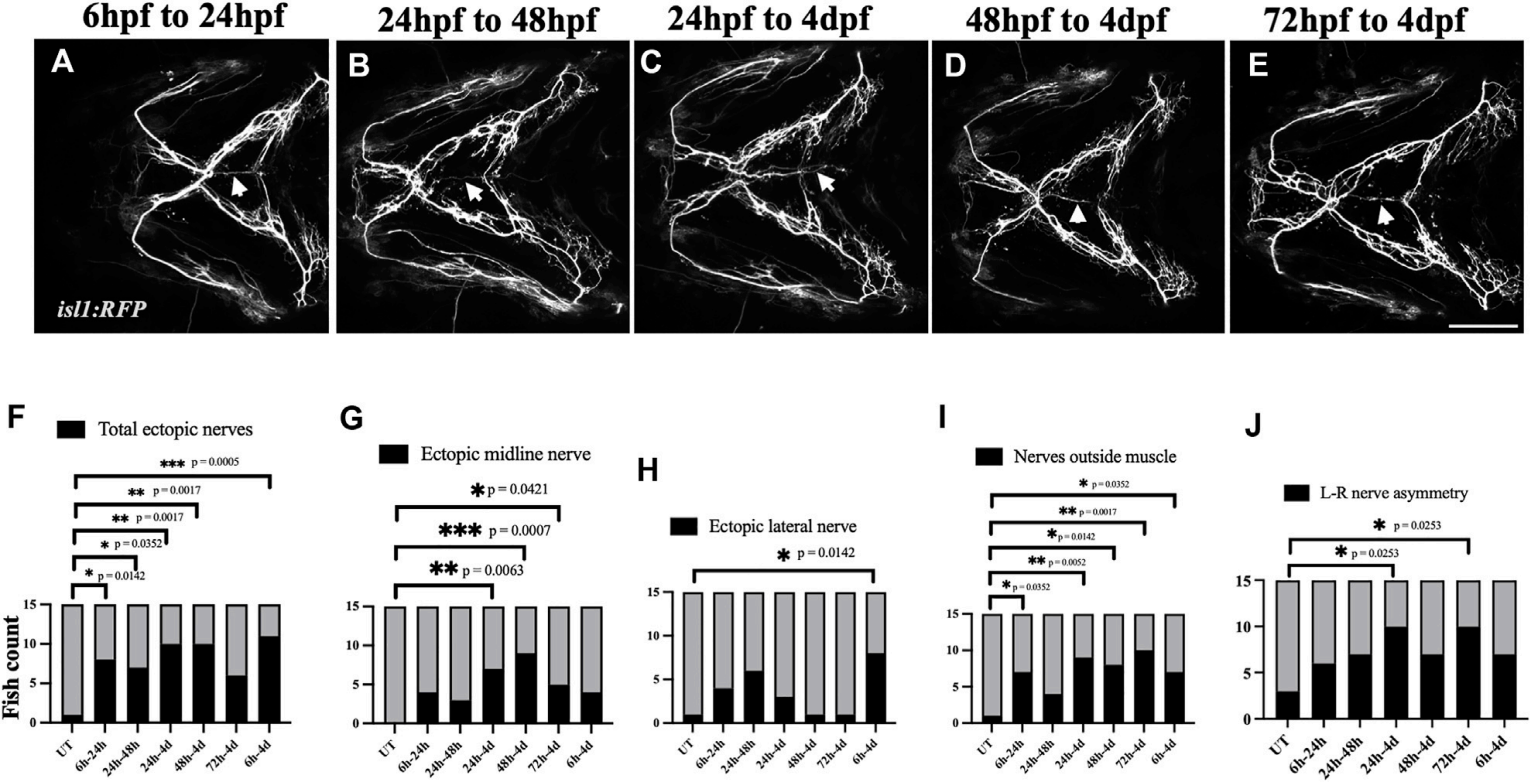
Ethanol-induced nerve defects occur at the time of muscle innervation **(A–E)** 1% ethanol exposure between 6hpf-24hpf, 24hpf-48hpf, 24hpf-4dpf, 48hpf-4dpf and 72hpf-4dpf result in ectopic nerve defects. Arrows indicate ectopic nerves along the medial axis. **(F–J)** Number of ethanol-exposed *isl1:RFP* fish with total ectopic nerves, ectopic midline nerves, ectopic lateral nerves, nerve positioned outside the muscle, left-right nerve asymmetry (*n* = 15 in each group). The frequency of various nerve defects significantly increased in fish exposed to ethanol between 24 h-4 d and 48 h-4 d. (F, G) The frequency of ectopic nerve defects particularly increased in fish exposed to ethanol between 48 h and 4 d. (h/hpf-hours post fertilization, d-days post fertilization). * = *p* < 0.05; ** = *p* < 0.01; *** = *p* < 0.001. Scale bar = 100 um.

Next, we investigated the developmental time window at which the cranial nerves are most sensitive to ethanol. We exposed *isl1:RFP* embryos to 1% ethanol at various developmental time windows: 6hpf-24hpf, 24hpf-48hpf, 24hpf-4dpf, 48hpf-4dpf & 72hpf-4dpf (Figures 9A–E). We found that the frequency of total ectopic nerves and ectopic midline nerves increase in embryos exposed to ethanol between 48hpf-4dpf (Figures 9F, G). Interestingly, the cranial muscles emerge and elongate after 52hpf (Schilling and Kimmel, 1997). This shows, the developmental time of cranial muscle elongation coincides with the time window at which the cranial nerves are most sensitive to ethanol. Collectively, these results suggest that ethanol does not cause early misrouting defects in cranial nerve, but rather during muscle innervation.

### 2.6 Effect of ethanol on muscles and nerve can be independent of one another

Our analysis on embryos exposed to 1% ethanol demonstrate that ectopic muscle defects are not associated with connective tissue and skeletal defects. This indicates that the effect of ethanol on these tissue types is distinct. However, cranial muscles and motor nerves are both sensitive to 1% ethanol and axonal defects occur during the time of target selection and innervation. Given their close association, it is possible that the muscle and neural defects are related. Using the *isl1:GFP;unc:mCherry* double transgenic line, we investigated if ethanol can concurrently induce ectopic muscle and ectopic nerve defects.

**FIGURE 10:**
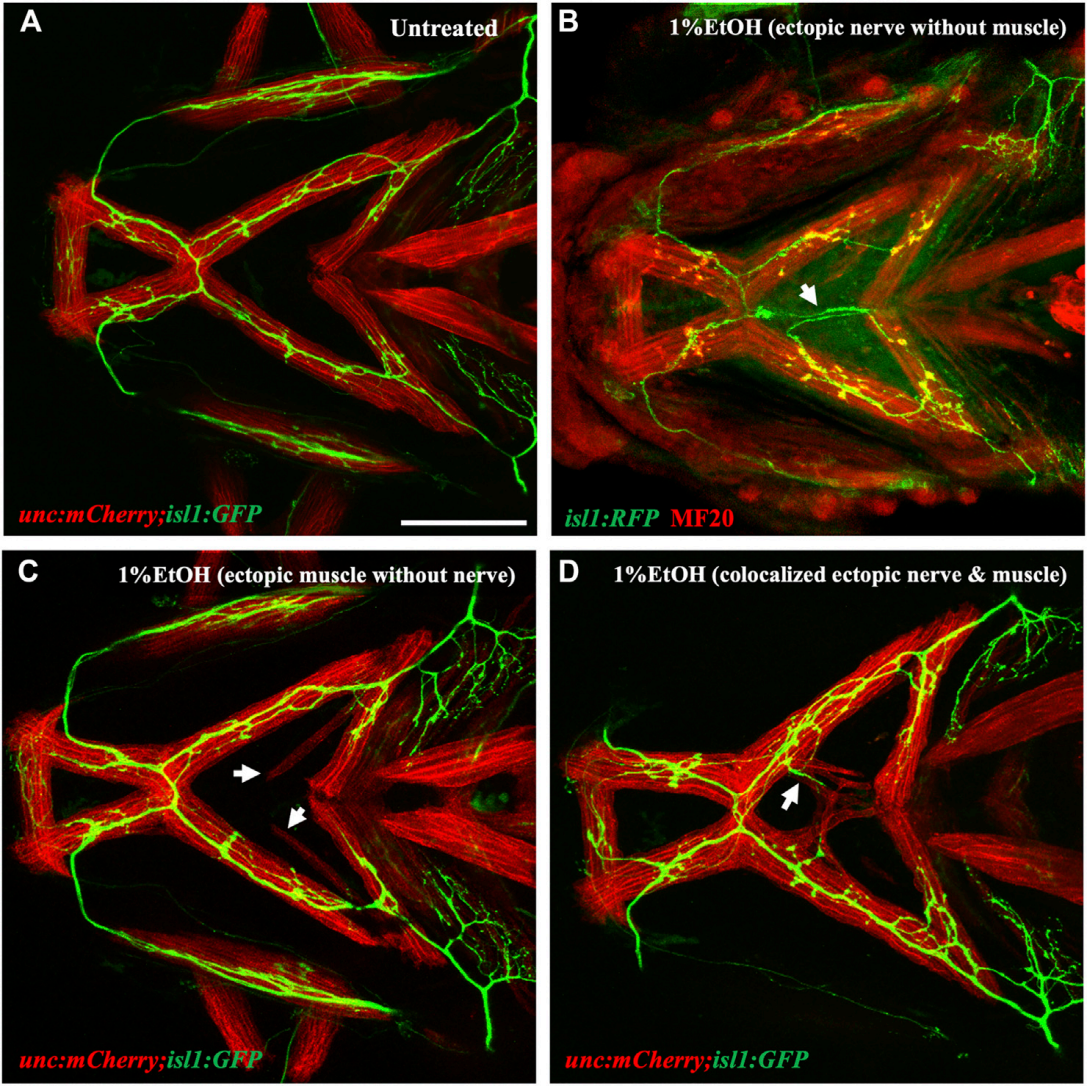
Ectopic nerve defects are independent of ectopic muscle defects **(A)** Untreated *isl1:GFP;unc:mCherry* showing muscles in red and motor nerves in green. **(B)** Ethanol-exposed *isl1:RFP* fish immunolabeled with anti-RFP (in green) and MF20 (in red). Arrow indicates ectopic nerve without ectopic muscle. **(C)** Ethanol-exposed *isl1:GFP;unc:mCherry* fish with ectopic muscle fibers without motor innervation. Arrows indicate ectopic muscle fibers. **(D)** Ethanol-exposed *isl1:GFP;unc:mCherry* fish with coexisting ectopic nerve and ectopic muscle indicated by the arrow. Scale bar = 100 um.

Embryos were exposed to 1% ethanol from 6hpf to 4dpf and analyzed for muscle and nerve defects at 4dpf. Interestingly, we found ectopic nerves which did not innervate muscle fiber and were located far-away from the muscle territory (Figure 10B). In other cases, we found several fish with ectopic muscle fibers completely lacking motor innervation (Figure 10C). There were also a few fish with ectopic muscle fibers supplemented with motor innervations having colocalized ectopic muscle and nerve (Figure 10D). We scored the number of defects in each category (note, any individual fish can have more than one defect) and found that 46.67% fish showed no defects, 46.67% fish showed ectopic muscles without motor innervation, 53.33% fish had ectopic nerves without associated ectopic muscle and 26.66% with colocalized ectopic nerves and muscle. These results suggest that there is no association between the incidences of ectopic nerves and ectopic muscles despite the fact that the defects are coincident in developmental time and location. Also, unlike muscles, the cranial nerves were not rescued by immobilization with MESAB (Supplementary Figure S3). This further substantiates that ethanol-induced muscle and nerve defects are unlinked to one another.

### 2.7 Ethanol causes reductions in the postsynaptic terminal

**FIGURE 11:**
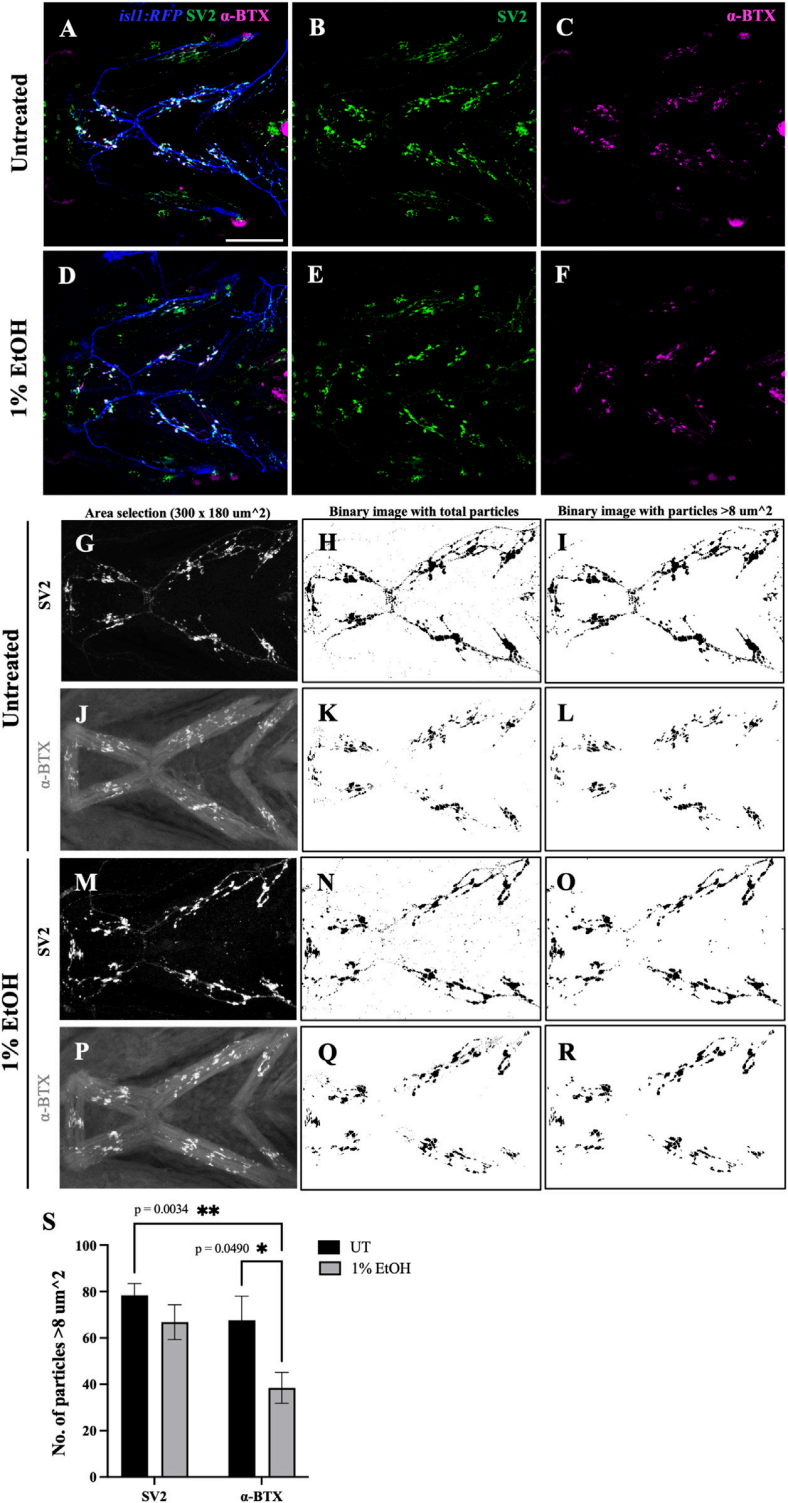
Number of postsynaptic receptors are reduced in ethanol-exposed fish **(A–F)** Immunostained neuromuscular junctions (NMJs) composed of presynaptic terminals (labeled with SV2) and postsynaptic acetylcholine receptors (labeled with α-BTX) in untreated **(A–C)** and ethanol-exposed **(D–F)** *isl1:RFP* fish. A and D are merged images with motor neurons (in blue), SV2 (in green) and α-BTX (in magenta). **(G–R)** Quantification of presynaptic and postsynaptic terminals in untreated **(G–L)** and ethanol-exposed **(M–R)** fish. NMJs were counted for Intermandibularis anterior, Intermandibularis posterior and Interhyal muscles by selecting an area of 300x180 um^2 as shown in G, J, M and P. H, K, N and Q show total number of particles after segmentation. I, L, O and R show particles with sizes greater than 8-micron square. **(S)** Number of α-BTX labeled particles >8 um^2 significantly reduced in ethanol-exposed fish. There was no significant change in the number of SV2 labeled particles. * = *p* < 0.05; ** = *p* < 0.01. Scale bar = 100 um.

The vertebrate craniofacial muscles are innervated by the cranial motor neurons residing in the vertebrate hindbrain (Guthrie, 2007). The communication between the cranial motor neurons and the muscle fibers are mediated through specialized synaptic association called neuromuscular junctions (NMJs). NMJs comprise of the 1) presynaptic nerve terminals formed by the axonal end, 2) the synaptic cleft where the neurotransmitter acetylcholine (ACh) is released and 3) the postsynaptic muscle endplate with aggregated acetylcholine receptors (AChRs) (Jing et al., 2010). To test if ethanol disrupts NMJs, we investigated the distribution of synaptic junctions in the branchial musculature of zebrafish exposed to 1% ethanol from 6hpf to 4dpf. We immunostained untreated and ethanol-exposed *isl1:RFP* fish at 4dpf with Synaptic vesicle-2A (SV2) and alpha-Bungarotoxin (α-BTX) antibodies to label the pre- and post-synaptic terminals, respectively (Figure 11).

Not surprisingly, we observed that ectopic muscles lacking motor innervation did not develop NMJs (Supplementary Figure S4C). Similarly, there were no presynaptic terminals formed when the ectopic nerve was not accompanied by an ectopic muscle (Supplementary Figure S4D) reiterating the reciprocal interaction required between the nerve and muscle for synaptogenesis. However, where both ectopic nerve and muscle were present in the same location, ectopic NMJs were induced (Supplementary Figure S4I).

To examine the effect of ethanol on NMJs within normally localized muscle, we quantified the number of NMJs in the Intermandibularis anterior, Intermandibularis posterior and the Interhyal muscles (Figures 11G–R, Supplementary Table S2). In untreated fish the number of pre- and post-synaptic terminals were balanced, as expected (Figure 11S). However, in ethanol-exposed fish there was a significant reduction in the number of postsynaptic terminals relative to presynaptic terminals (Figure 11S). This finding demonstrates that ethanol disrupts synaptogenesis in the developing head.

## 3 Discussion

Using a variety of transgenic lines, we studied the effect of ethanol exposure on craniofacial integration and assembly. As would be expected from previous research (Muralidharan et al., 2013; Luo, 2014; Caputo et al., 2016; Almeida et al., 2020), we found that neural tissue was more sensitive to ethanol than the skeleton. We note that we were able to identify a previously uncharacterized facial defect in the ethanol-exposed fish, altered basihyal shape. We suspect that this is due to the higher resolution confocal microscopy used in imaging the transgenic fish.

Our findings further demonstrate that the craniofacial musculature is more sensitive to ethanol than the skeleton. We also find that the varied ethanol-induced cranial defects are largely independent of one another. For instance, we found no association between the incidence of ectopic muscles and ectopic nerves despite their spatial and temporal overlap. The ectopic muscles were partially rescued by immobilizing the fish in MESAB/Tricaine. However, immobilization did not rescue the ectopic nerve defects. These findings demonstrate that the same amount and duration of ethanol exposure can have separate and independent effects on different tissues even when they are closely linked in developmental space and time.

### 3.1 Ethanol and musculoskeletal attachment

The cranial neural crest cells (CNCs) play a key role in the organization of the craniofacial complex. The patterning and integration of the mesoderm-derived muscle onto the primarily CNC-derived cranial skeleton is directed by the CNCs (Rinon et al., 2007; McGurk et al., 2017; Schneider, 2018; Ziermann et al., 2018). Ethanol is known to induce cell death and migratory defects in CNCs as demonstrated by experiments in zebrafish, chick, and mouse (Chen et al., 2000; Smith et al., 2014; Kiecker, 2016; Eason et al., 2017). Because of this, ethanol could disrupt the craniofacial complex as a whole *via* the neural crest. The dosage of ethanol used in this study, however, does not elevate cell death in the neural crest (McCarthy et al., 2013), suggesting that additional mechanisms are at play.

We found that ethanol can induce ectopic muscle fibers and can mislocate the myotendinous attachment at the mylohyoid junction. We could partially rescue the ectopic muscle fiber defect by anaesthetizing the fish suggesting these ectopic muscles resulted from fiber detachment and are not due to an underlying patterning defect. These ectopic fibers were never associated with a tendon. Interestingly, ethanol intake in adult rats results in abnormal tenocyte morphology and disorganized collagen during tendon healing (Hapa et al., 2009). This is consistent with our observation which suggests that ethanol exposure could be affecting tendon integrity allowing muscle fibers to detach from their initial site of attachment. It is most likely that the defect is in the attachment of muscle to tendon, rather than the tendon insertion site to the skeleton, as the latter is never disrupted. Further work is necessary to understand if and how ethanol affects tendon function and strength.

### 3.2 Ethanol and motor nerves

We found profound alterations to motor axon projections in ethanol-exposed fish. These defects occurred as the axons reached their muscle targets and do not appear to associate with any early pathfinding defects. The two major classes of defects were ectopic fibers and defasciculation. While we do not know the cause of these defects, both could be due to reduced cell-cell adhesion.

L1 is a member of the immunoglobin superfamily of cell adhesion molecules that is strongly implicated in FASD (Bearer, 2001). The L1 adhesion molecule has several known functions, including fasciculation (Wiencken-Barger et al., 2004). Ethanol is known to inhibit neural cell-cell adhesion in culture (Charness et al., 1994). This effect is, at least partly, due to ethanol binding the L1 adhesion molecule (Dou et al., 2013) and the redistribution of L1 in lipid rafts (Tang et al., 2011). L1 regulates fasciculation in zebrafish (Wolman et al., 2007), albeit the effects on branchiomotor neurons have not been characterized. It will be of interest to determine if the axonal defects characterized here are L1-dependent.

### 3.3 Ethanol and the neuromuscular junction

The skeletal muscle and the innervating motor nerve share a close anatomical association. This study and others show that ethanol can induce morphological defects in muscles (Nyquist-Battie et al., 1987; Li et al., 2007) and motor innervation (Sylvain et al., 2010). Communication between motor neurons and muscle fibers occurs at neuromuscular junctions (NMJs) (Brehm and Wen, 2019). Here, we found that ethanol specifically reduced the number of postsynaptic termini. While we did not analyze any potential physiological defects in these fish, exposure of zebrafish to higher concentration of ethanol has been shown to cause motor deficits (Sylvain et al., 2010).

A series of reciprocal interactions between the innervating axonal fiber and the muscle endplate is necessary to induce NMJs in their precise locations. The Agrin-muscle-specific kinase (MUSK)- low-density lipoprotein receptor-related protein 4 (Lrp4) signaling pathway plays a key role in establishing the NMJ, particularly clustering AChRs in the postsynaptic termini (Jing et al., 2010). Ethanol has been shown to reduce agrin-induced acetylcholine receptor clustering in C2C12 cells (Owen et al., 2010). During eye and brain development, ethanol disrupts Agrin function (Zhang et al., 2011). Thus, ethanol may disrupt the initiation of the signaling pathway required to cluster AChRs in the postsynaptic terminus.

It is also possible that the effect of ethanol is directly on postsynaptic terminus. Chronic ethanol exposure in rats can downregulate the expression of the acetylcholine receptor alpha-subunit gene at the transcriptional or posttranscriptional level (Held et al., 1991). Ethanol can also directly interact with the alpha-subunit of AChR (Noori et al., 2018). These studies further show that the postsynaptic AChR clustering is particularly sensitive to ethanol. Further study is required to elucidate the detailed mechanism underlying ethanol-induced defective synaptogenesis.

Collectively, our study carried out a comprehensive examination of the effect of ethanol on integration and assembly of the vertebrate craniofacial complex. We demonstrate that the effect of ethanol on each tissue type is distinct and largely independent from one another. The soft tissues such as nerves and muscles are more ethanol sensitive than hard tissues such as bones and cartilages. Characterizing soft tissue defects at low ethanol concentrations which do not induce skeletal defects may provide improved identification criteria for early clinical assessment for non-dysmorphic FASD cases. We also show that ethanol disrupts the craniofacial integration particularly affecting the neuromuscular associations. These defects may underlie the motor functioning of the facial musculature linked to speech and swallowing defects in children with FASD.

## 4 Material and methods

### 4.1 Zebrafish husbandry and transgenic lines

All fish lines used in this study were housed and raised in the University of Texas at Austin based on established protocols (Westerfield, 2000) approved by the Institutional Animal Care and Use Committee. Embryos were staged based on Kimmel et al., 1995. The *Tg(sox10:Kaede)zf393* line (Dougherty et al., 2013) was used to label the branchial cartilages, this line has been referred to as *sox10:Kaede* throughout the text. The *Tg(isl1:GFP)rw0* line (Higashijima et al., 2000) and *Tg(isl1-hsp70l:mRFP)fh1* line (Grant and Moens, 2010) were used to visualize the branchiomotor neurons. The *Tg(isl1:GFP)rw0* and *Tg(isl1-hsp70l:mRFP)fh1* lines have been referred to as *isl1:GFP* and *isl1:RFP* in the text. The *Tg(-0.5unc45b:mCherry)au101* line (McGurk et al., 2017) labels the muscles, this line has been referred to as *unc:mCherry* in the text. The tg*(scxa:mCherry)fb301* line (McGurk et al., 2017) was used to label the tendons, this line has been referred to as *scxa:mCherry* in the text.

### 4.2 Ethanol exposure

Zebrafish embryos were exposed to varying ethanol concentrations at different time points during development. To study the effect of ethanol exposure on craniofacial skeleton, muscle, connective tissue and cranial nerve; ethanol concentration of 1% volume by volume (v/v) in embryo media (EM) was used. The embryos were raised in the ethanol-containing media from 6hpf to 4dpf. All experiments were repeated with at least two independent clutches with *n* = 15 each in ethanol-exposed and untreated group. Data from two separate experiments were combined to get *n* = 30 in each group for statistical analyses shown in this paper. Most studies have found that the tissue-ethanol concentration is ∼25%–30% of the media ethanol concentration. 1% ethanol in media corresponds to ∼51.3 mM tissue ethanol concentration. This amount equates to binge drinking in humans (Lovely et al., 2014). For the dose response study, embryos were exposed to ethanol concentrations 0.25%, 0.5%, 0.75%, 1% v/v in EM from 6hpf to 4dpf. The dose response study was repeated once with a single clutch of *isl1:RFP* fish with *n* = 15 in each group. To study the effect of ethanol at different developmental time windows embryos were exposed to ethanol concentration of 1% v/v in EM between 6hpf-4dpf, 6hpf-24hpf, 24hpf-48hpf, 24hpf-4dpf, 48hpf-4dpf and 72hpf-4dpf. At the end of the exposure, ethanol-containing EM was replaced with ethanol-free EM, the embryos were allowed to develop in fresh EM until 4dpf. A single clutch of *isl1:RFP* embryos with *n* = 15 in each group was used in this study.

### 4.3 Alcian-Blue & Alizarin-red staining

Alcian-Blue & Alizarin-Red staining was performed on ethanol-exposed wild type AB and *sox10:Kaede* fish using the protocol described in Walker and Kimmel, 2007. The fish were imaged in 50% glycerol using Zeiss AxioPhot, ×10 objective lens.

### 4.4 Whole-mount (WM)- immunohistochemistry (IHC)

4dpf-fish were fixed overnight in 4% paraformaldehyde and washed in 1X PBS containing 1%BSA, 1% DMSO, 0.5% Triton X-100. Primary antibodies were incubated overnight at 4°C in 5% normal goat serum, 1%BSA, 1% DMSO, 0.5% Triton X-100. For muscle and connective tissue staining MY1H (DSHB MF20 at 2 ug/mL) and Thbs4b (GeneTex-GTX125869 at 1:500 dilution) antibodies were used. To investigate the neuromuscular junctions, Synaptic vesicle glycoprotein 2 A (DSHB SV2 at 5 ug/mL) and α-Bungarotoxin (Alexa Fluor 647 conjugated-B35450 at 1:250 dilution) were used to label the presynaptic and postsynaptic termini, respectively. Anti-RFP (DSHB) and anti-mCherry (DHSB 3A11) at 2 ug/mL were used to label nerves and connective tissues in *isl1:RFP* and *scxa:mCherry* fish. Anti-Kaede antibody (MBL M106-3M) at 1:250 dilution was used to label skeleton in *sox10:Kaede* fish*.* Secondary antibodies (1:500 dilution) were also incubated overnight at 4°C in 5% normal goat serum, 1%BSA, 1% DMSO, 0.5% Triton X-100. Following incubation, antibody solution was removed, the fish were washed three times in 1X PBSTx (0.5% Triton X-100).

### 4.5 Confocal microscopy

Fish were anesthetized using MESAB (MS 2222/Tricaine) and mounted in 0.2% agarose and 3% Methyl Cellulose. Bidirectional confocal Z-stacks were acquired using Zeiss LSM 710 & LSM 980. Images were processed using ImageJ/FIJI.

### 4.6 ImageJ analysis for quantification of neuromuscular junctions

Ethanol-exposed and untreated embryos immunolabeled with SV2 and α-BTX antibodies were imaged using Zeiss LSM 980 using ×20 objective lens. Confocal stacks were Z-projected with maximum intensity. The area selection tool was used to crop out a region of 300 x 180 um^2 (= 750 x 450 in pixel units) containing Intermandibularis anterior, Intermandibularis posterior and Interhyal muscles. The rolling ball background subtraction plugin was used to correct the uneven background illumination by setting a radius of 50.0 pixels (pixel size = 0.4 um in length and breadth) for both SV2 and α-BTX channels. The images were segmented by setting a specific threshold cutoff value for SV2 and α-BTX channels. Global thresholding was performed on SV2 and α-BTX channels and converted to binary images. Watershed plugin was applied to the binary images to separate connected particles. The number of particles in the segmented image were counted using the Analyze particle tool with circularity 0.00-1.00 and size 50 um^2-infinity in pixel units (= 8 um^2 in scaled units). The number of SV2 and α-BTX labelled particles with area greater than 8-micron square in untreated and ethanol-exposed fish were plotted. This experiment was repeated twice to get a final count of *n* = 14 in each group.

### 4.7 Time-lapse imaging

Time lapse imaging was performed based on established protocol (McGurk et al., 2014). To investigate early cranial nerve routing *sox10:Kaede;isl1:RFP* embryos were exposed to 1% ethanol from 6hpf to 24hpf. At 24hpf, untreated as well as ethanol-exposed embryos were anaesthetized in MS 2222/Tricaine and mounted laterally in 0.2% agarose and 3% Methyl Cellulose. The fish were imaged on a temperature regulated stage set at 28.5°C. Bidirectional confocal z-stacks were collected using Zeiss LSM 980 with time interval of 12 min until 48hpf. Images were processed using ImageJ/FIJI.

### 4.8 Live alizarin staining

72hpf *sox10:Kaede* embryos were transferred to embryo medium containing 25 ug/mL Alizarin red and 12.5 mM HEPES buffer and incubated in dark at 28.5°C. The embryos were live imaged at 4dpf.

### 4.9 Muscle rescue experiment by immobilization

Embryos were exposed to 1% ethanol at 6hpf and allowed to develop at 28.5°C. At 52hpf, fish were dechorionated and transferred to embryo medium containing 1% ethanol and 0.075 mg/mL MS 2222/Tricaine; and allowed to develop at 28.5°C. The fish were imaged at 4dpf to characterize muscle and nerve defects. This experiment was repeated once with a single clutch of *unc:mCherry* fish with *n* = 15 in each group.

### 4.10 Statistical analysis

Fisher’s exact test of independence was used to examine if ethanol-induced defects were statistically significant. One-way ANOVA with Tukey’s multiple comparisons correction was used for analysis of defasciculation defects in untreated, 0.25%, 0.5%, 0.75%, 1% ethanol-exposed fish. Two-way ANOVA with Tukey’s multiple comparisons correction was used for comparison of the number of neuromuscular junctions in the ethanol-exposed and untreated fish. For all analyses, data are presented as means ± SEM and *p* < 0.05 was considered statistically significant. GraphPad Prism nine was used for all statistical analyses.

## Data Availability

The original contributions presented in the study are included in the article/Supplementary Material, further inquiries can be directed to the corresponding author.
