# Supplementary material for: Embryonic ethanol exposure disrupts craniofacial neuromuscular integration in zebrafish larvae
Source: Front Physiol. 2023 Feb 7;14:1131075. doi: 10.3389/fphys.2023.1131075 (PMC9941677; doi:10.3389/fphys.2023.1131075)
Supplement: Supplementary file 4 [file DataSheet1.DOCX]

Supplementary Material

Embryonic ethanol exposure disrupts craniofacial neuromuscular integration in zebrafish larvae

Ritika Ghosal^1^, Gissela Borrego-Soto^1^, Johann K. Eberhart^1*^

^1^Department of Molecular Biosciences, University of Texas at Austin, Austin, Texas, USA

*** Correspondence: Johann K. Eberhart**

eberhart@austin.utexas.edu

**Supplementary Figure 1: Anesthetizing ethanol-exposed fish before muscle attachment and extension partially rescues ectopic muscle defects**

(A) Schematic showing time-windows for ethanol and MESAB treatments. (B) Representative images showing ventral branchial muscles in untreated, 1% ethanol-exposed, and 1% ethanol-exposed + 0.075mg/ml MESAB-treated fish. Arrows show ectopic muscle fibers. (C) Number of *unc:mCherry* fish with ectopic muscle fibers in untreated, 1% ethanol-exposed and 1% ethanol-exposed + 0.075mg/ml MESAB-treated groups. Anesthetizing ethanol-exposed fish reduced the frequency of ectopic muscles. * = p < 0.05. Scale bar = 100um.

**Supplementary Figure 2: Mylohyoid junction is not attached to basihyal**

(A) Orthogonal sections through mylohyoid junction (MHJ) in *sox10:Kaede;scx:mCherry* do not show any physical association between MHJ tendon and basihyal. Arrows indicate a physical gap between MHJ (in red) the basihyal (in green) along the YZ and XZ planes. (B) Orthogonal sections through sternohyoid tendon (SHT) show SHT is attached to hypohyal cartilage, arrows indicate the attachment between SHT and hypohyal. (C) Single Z-slice showing no overlap of MHJ tendon with any skeletal element. (D) Snapshot from 3D-projected Z-stack (Movie 1) showing a gap between MHJ and basihyal.

**Supplementary Figure 3: Anesthetizing ethanol-exposed fish does not rescue ectopic nerve defects**

(A-C) Representative images showing motor innervations in ventral branchial muscles in untreated, 1% ethanol-exposed and 1% ethanol-exposed + 0.075mg/ml MESAB-treated fish. Arrows indicate ectopic nerves (D) Number of *isl1:RFP* fish with ectopic nerves in untreated, 1% ethanol-exposed and 1% ethanol-exposed + 0.075mg/ml MESAB treated groups. * = p < 0.05; ** = p <0.01. Scale bar =100um.

**Supplementary Figure 4: Ethanol-induced ectopic muscles and nerves can induce ectopic neuromuscular junctions.**

(A-C) Synaptic vesicle 2A (SV2) and alpha-Bungarotoxin (α-BTX) colabeled *isl1:RFP* fish with ectopic muscle fibers without motor innervation (indicated by arrows in B & C). Arrow in C shows no ectopic neuromuscular junctions (NMJs) in the ectopic muscle fiber without motor innervation. (D-F) SV2 & α-BTX colabeled *isl1:RFP* fish with ectopic nerve without ectopic muscle. Arrow in D shows ectopic nerves along the medial axis (in blue). Arrows in F show a few ectopic presynaptic vesicles (in red) formed along the part of the ectopic nerve situated close to the muscle. However, the region of the nerve which is distant from the muscle did not form presynaptic terminals. (E, F) No postsynaptic receptors formed along the ectopic nerve. (G-I) Ectopic NMJs were induced when both ectopic nerve and ectopic muscle coexisted in the same space. Arrow in G shows presynaptic terminals induced along an ectopic midline nerve. Arrow in H shows postsynaptic receptors in ectopic muscle fiber innervated by an ectopic nerve. Arrow in I shows ectopic NMJs.

**Supplementary table 1:** ANOVA P-value summary for analysis shown in Figure 7G. * = p < 0.05; ** = p <0.01; *** = p < 0.001; **** = p < 0.0001.

**Supplementary table 2:** ANOVA P-value summary for analysis shown in Figure 11S. * = p < 0.05; ** = p <0.01; *** = p < 0.001; **** = p < 0.0001.
